# Looking though the Past, Present and Future of TMS-EEG

**DOI:** 10.1192/j.eurpsy.2023.1956

**Published:** 2023-07-19

**Authors:** S. N. Martins, C. Romano, B. Ribeiro

**Affiliations:** 1 Centro Hospitalar Tâmega e Sousa, Penafiel

## Abstract

**Introduction:**

Psychiatry has been diagnosing its pathologies through the evaluation of the symptoms reported by patients, relying on a few complementary exams to exclude organic causes. Studies about transcranial magnetic stimulation and electroencephalography (TMS-EEG) are bringing, from a clinical point of view, crucial information to characterize the different pathophysiological biomarkers of the psychiatric diseases, leading not only to the evolution of diagnosis, but also to an improved, more individualized treatment.

**Objectives:**

Characterizing the state of the art of TMS-EEG and its use in psychiatric diagnosis and treatments of different diseases.

**Methods:**

We undertook a narrative literature review by performing a search on PubMed for English-written articles from the last 10 years. The query used was “TMS-EEG”; “TMS-EEG” AND “Schizophrenia” OR “Major Depressive Disorder” OR “Bipolar Disorder”.

**Results:**

Transcranial magnetic stimulation (TMS) is a safe and reliable method of non-invasive brain stimulation that allows for the local activation of cortical areas through electromagnetic induction. When combining this method with electroencephalography (EEG), it enables the underlying mechanisms of brain diseases.

TMS is a powerful therapeutic technic in Major Depressive Disorder (MDD). The literature refers to an enhanced N45 and N100 amplitude, which indicates a baseline cortical inhibition that can indicate a depressed state, which can be used as a clinical biomarker to evaluate TMS treatments.

In Schizophrenia (SCZ), TMS-EEG reveals a decreased cortical inhibition and excitation. Indices of inhibition and excitation reductions were also related to cognitive deficits.

The current studies regarding Bipolar Disorder (BD) are not so consistent, revealing that there are shared neural pathways with MDD and SCZ. This is a pathology often misdiagnosed with MDD, so biomarkers would help to diagnose BD earlier and improve its prognostic.

**Conclusions:**

TMS-EEG can be used to provide more accurate neural targets, leading to more powerful and personalized interventions in psychiatric disorders, as well as more accurate diagnoses.

As for future studies, it would be relevant to assess not only TMS treatment effects, but also pharmacological results in these different pathologies.

**Disclosure of Interest:**

None Declared

